# Functional development of the adult ovine mammary gland—insights from gene expression profiling

**DOI:** 10.1186/s12864-015-1947-9

**Published:** 2015-10-05

**Authors:** Amy M Paten, Elizabeth J Duncan, Sarah J Pain, Sam W Peterson, Paul R Kenyon, Hugh T Blair, Peter K Dearden

**Affiliations:** Laboratory for Evolution and Development, Genetics Otago, Department of Biochemistry, University of Otago, P.O. Box 56, Dunedin, Aotearoa New Zealand; International Sheep Research Centre, Institute of Veterinary, Animal and Biomedical Sciences, Massey University, Palmerston North, Aotearoa New Zealand; Gravida; National Centre for Growth and Development, Auckland, New Zealand

**Keywords:** Sheep, Mammary gland, RNA-seq, Development, Lactation

## Abstract

**Background:**

The mammary gland is a dynamic organ that undergoes dramatic physiological adaptations during the transition from late pregnancy to lactation. Investigation of the molecular basis of mammary development and function will provide fundamental insights into tissue remodelling as well as a better understanding of milk production and mammary disease. This is important to livestock production systems and human health.

Here we use RNA-seq to identify differences in gene expression in the ovine mammary gland between late pregnancy and lactation.

**Results:**

Between late pregnancy (135 days of gestation ± 2.4 SD) and lactation (15 days post partum ± 1.27 SD) 13 % of genes in the sheep genome were differentially expressed in the ovine mammary gland. In late pregnancy, cell proliferation, beta-oxidation of fatty acids and translation were identified as key biological processes. During lactation, high levels of milk fat synthesis were mirrored by enrichment of genes associated with fatty acid biosynthesis, transport and lipogenesis. Protein processing in the endoplasmic reticulum was enriched during lactation, likely in support of active milk protein synthesis. Hormone and growth factor signalling and activation of signal transduction pathways, including the JAK-STAT and PPAR pathways, were also differently regulated, indicating key roles for these pathways in functional development of the ovine mammary gland. Changes in the expression of epigenetic regulators, particularly chromatin remodellers, indicate a possible role in coordinating the large-scale transcriptional changes that appear to be required to switch mammary processes from growth and development during late pregnancy to synthesis and secretion of milk during lactation.

**Conclusions:**

Coordinated transcriptional regulation of large numbers of genes is required to switch between mammary tissue establishment during late pregnancy, and activation and maintenance of milk production during lactation. Our findings indicate the remarkable plasticity of the mammary gland, and the coordinated regulation of multiple genes and pathways to begin milk production. Genes and pathways identified by the present study may be important for managing milk production and mammary development, and may inform studies of diseases affecting the mammary gland.

**Electronic supplementary material:**

The online version of this article (doi:10.1186/s12864-015-1947-9) contains supplementary material, which is available to authorized users.

## Background

The mammary gland, and the physiological control of lactation, evolved as a vital part of the mammalian reproduction strategy [1, 2]. Milk provides an essential source of nutrients to newborn mammals, as well as immune factors, including anti-microbial, anti-inflammatory and immune-modulatory agents, that offer protection against infections, and have beneficial effects on intestinal flora and gut health [2, 3]. Human epidemiological studies provide evidence that breast milk plays a role in protection against gastrointestinal and respiratory tract infections in infants, and in programming metabolism and disease later in life [4–7]. Additionally, humans have long exploited the production of milk by domestic ruminants for the manufacture of dairy products, making milk an important part of human nutrition. Milk production is also important in pastoral livestock systems for the production of meat and fibre. In such systems, milk produced by the dam is the sole source of nutrients for newborn offspring and may influence their survival, growth to weaning, and future productive performance [8–10]. The ability to manipulate lactation performance is an area of increasing interest, and knowledge of the biological pathways and mechanisms that govern mammary gland development and lactation is commercially important.

The mammary gland displays a high level of developmental plasticity, able to undergo repeated cycles of growth, differentiation, and regression, coordinated by the reproductive state [11]. Mammary development follows the reproductive cycle and is regulated by endocrine hormones [11–13]. The dynamic nature of the mammary gland makes it an ideal model for studying molecular regulation of development and cellular differentiation. Mammary glands are comprised of two main tissue components: the parenchyma and the stroma [14]. The parenchyma contains the functional secretory and ductal tissue. The mammary stroma, or fat pad, contains the supportive tissue, including connective tissue, fibroblasts, adipose tissue, nerve tissue, and endothelial cells associated with blood vessels and lymph vessels [13, 14].

While mammogenesis is initiated during embryonic life, the majority of mammary development occurs post-natally, particularly during pregnancy when there is marked expansion of the lobulo-alveolar network (clusters of alveoli, which are spherical structures, comprised of secretory epithelial cells surrounding a central lumen, and basal myoepithelial cells which contract to allow milk let-down) [15]. The number of mammary epithelial cells is correlated to milk yield [16–19], thus establishment of secretory tissue during this developmental stage is critical for subsequent lactational performance. Prior to parturition, functional differentiation of epithelial cells is initiated, referred to as lactogenesis stage 1 [20]. This stage is characterised by cytological changes as well as increasing expression of genes encoding milk proteins such as caseins and beta-lactoglobulin [20–22]. The second stage of lactogenesis (lactogenesis stage two or secretory activation phase) is initiated at parturition and involves the closing of tight junctions between alveolar epithelial cells and further increases in expression of milk proteins, including *LALBA* (alpha-lactalbumin) which promotes lactose synthesis and associated increases in milk volume [21, 23].

In adult sheep, mammary development is essentially complete by parturition and there is only limited mammary growth during early lactation [24]. This is in contrast to the more extensive continued development observed in the mammary glands of litter bearing species, including rodents [25], pigs [26], and some dairy cows and goats [16, 27]. During early lactation, cellular metabolic activity and nutrient transport increases to provide substrates for synthesis of milk components [28].

Such coordinated regulation of developmental and functional events is likely mediated by large-scale changes in gene expression [29, 30]. While the structural changes, and roles of hormones, in events from late pregnancy to lactation are well known [29, 31–33], little is known about the underlying molecular mechanisms that regulate differentiation of the mammary epithelium to a secretory phenotype.

Here we use RNA-seq to examine global gene expression in sheep mammary glands during the transition from late pregnancy to lactation providing insights into the physiological and metabolic adaptations that occur during this transition. Understanding the regulation of this transition is essential to our ability to intervene in mammary development and disease, and to manipulate lactation outcomes important to animal production and human nutrition.

## Methods

### Animals and sample collection and processing

The animal study was conducted at the Massey University Keeble Sheep and Beef farm, 5 km south of Palmerston North, New Zealand. The study was approved by the Massey University Animal Ethics Committee, Palmerston North, New Zealand.

Mammary tissue was sampled from 2-year-old, primiparous, Romney ewes during late pregnancy (*n* = 27, ewe age: 733.9 days ± 1.66 SD and 135 days of gestation ± 2.4 SD) and again during early lactation (*n* = 18, ewe age: 761.0 ± 2.11 SD days and 15 ± 1.27 SD days post partum). Mammary parenchymal tissue (30–50 mg) was collected with a needle biopsy (Bard® Magnum® reusable core biopsy gun and 12G, 10 cm core biopsy needles, Bard Biopsy Systems, AZ, USA) as described by Norgaard et al. [34]. Tissue samples were immediately frozen in liquid nitrogen, then stored at − 80 °C until RNA extraction.

Total RNA was isolated from mammary tissue samples using Trizol (Invitrogen, CA, USA) and purified using RNeasy mini kit (Qiagen, Netherlands). Genomic DNA contamination was eliminated via on-column digestion with DNase (Qiagen), as per the manufacturer’s protocol, and verified by RT-PCR. The concentration and quality of RNA was measured using a Nanodrop ND-1000 spectrophotometer (Thermo Scientific, MA, USA) and integrity was assessed using an Agilent 2100 Bioanalyzer (Agilent Technologies, CA, USA). Only RNA with RNA integrity numbers (RINs) above seven was used in this study. One μg of total RNA was used as template to perform cDNA synthesis using the SuperScript VILO cDNA Synthesis Kit (Invitrogen) as per the manufacturer’s protocol.

### RNA sequencing and data analysis

For RNA sequencing, we attempted to minimise the effects of individual variation between animals by pooling RNA, as described by Paten et al. [35]. Briefly, each pool contained a total of 2 μg of RNA, sampled from three randomly selected animals. Nine pools were generated for late pregnancy samples (*n* = 27) and six for lactation samples (*n* = 18).

RNA-seq data was generated from pooled RNA (as detailed above) using an Illumina Hi-Seq 2000 (GEO accession number GSE71424). Mapping of the 100 bp paired-end sequence reads to the *Ovis aries* genome (version 3.2) and analysis of expression data was carried out using CLC Genomics Workbench (CLC Bio). Read mapping statistics are supplied in Table S1 (Additional file 1: Table S1). Data were subjected to quantile normalization and RPKM (reads per kilobase (kb) per million mapped reads statistic (RPKM = total exon reads mapped/ mapped reads in millions × exon length in kb)) for each annotated gene (10,175 genes) were calculated. Differentially expressed genes were identified using the Baggerly test [36]. The Baggerly test is similar to a two sample *t*-test but the test statistic is weighted according to the number of reads in each sample [36]. *P*-values were corrected for multiple testing using the method of Benjamini and Hochberg [37]. Genes that had a corrected *P* value of less than 0.05 were considered to be differentially expressed (Additional file 2: Tables S2 and S3). Principal components and heat map analyses were performed to evaluate general patterns of variation in gene expression between treatment groups (Additional file 3: Figure S1). The findings of our RNA-seq data were validated by reverse transcription quantitative (RT-qPCR) analysis of a sub-set of 18 genes, expression was normalized against established reference genes for the ovine mammary gland [35]. The details of which are reported in Additional file 4.

### Gene ontology and pathways analyses

Analysis of the RNA-seq data provided a list of differentially expressed genes. In order to contextualise this with physiological and metabolic events occurring in the mammary gland during late pregnancy and lactation, DAVID [38, 39] was used to identify enriched gene ontology (GO) and perform KEGG (Kyoto Encyclopedia of Genes and Genomes) pathways analyses (Fisher’s exact *P*-value < 0.01, no correction for multiple testing was applied). One limitation to these analyses is that, at the time of this analysis, many sheep genes did not have assigned functional categories assigned to them. To overcome this, mouse orthologs of sheep genes were identified based on homolog gene annotation in the NCBI gene database (http://www.ncbi.nlm.nih.gov/gene). Gene lists consisting of genes more highly expressed during late pregnancy (Additional file 2: Table S2) and lactation (Additional file 2: Table S3) were submitted to DAVID (http://david.abcc.ncifcrf.gov/tools.jsp) and a background list was constructed consisting of all of the genes expressed in the ovine mammary gland for which a mouse gene ortholog could be identified (*n* = 6733). Functional annotation clustering was used to cluster similar GO terms together and results were ranked according to the Group Enrichment Score (the geometric mean (in-log scale) of member’s *P*-values in a corresponding annotation cluster). Functional annotation clusters were given an overall term which summarised the general theme of each cluster and any clusters with enrichment scores below 2.0 (*P* = 0.01) were discarded (Additional file 5: Tables S5 and S6).

## Results and discussion

We detected a total of 10,132 genes expressed in ovine mammary gland tissue during late pregnancy and 10,096 genes expressed during lactation (9622 genes expressed in both late pregnancy and lactation), which accounts for approximately 49 % of genes in the sheep genome. Unfortunately a number of these genes have not been functionally annotated, including 4717 genes that were either predicted or uncharacterised. Validation of a subset of these genes by RT-qPCR (Additional file 4: Figure S2) indicates that our RNA-seq data is high quality and biologically relevant.

### Highly expressed genes

A very small number of genes accounts for 60 % of all RNA-seq reads (30 and 24 genes in late pregnancy and lactation, respectively). The six most highly expressed genes during lactation (*BLG* (β-lactoglobulin), *CSN2* (β-casein), *CSN1S1* (α-S1-casein), *LALBA* (α-lactalbumin), *CSN3* (κ-casein), *GLYCAM1* (glycosylation dependent cell adhesion molecule-1) and *CSN1S2* (casein-α-S2)) have been reported to be highly expressed in cells derived from milk in other species (e.g. cattle [40] and human [41]) using RNA-sequencing. High-expression of some of these genes has been detected using other experimental approaches, including RT-qPCR (e.g. [42, 43]), supporting the conclusion that the ultra-high expression that we report for these genes reflects the biology of the mammary gland in late pregancy and lactation and is not an experimental artifact.

**Fig. 1:**
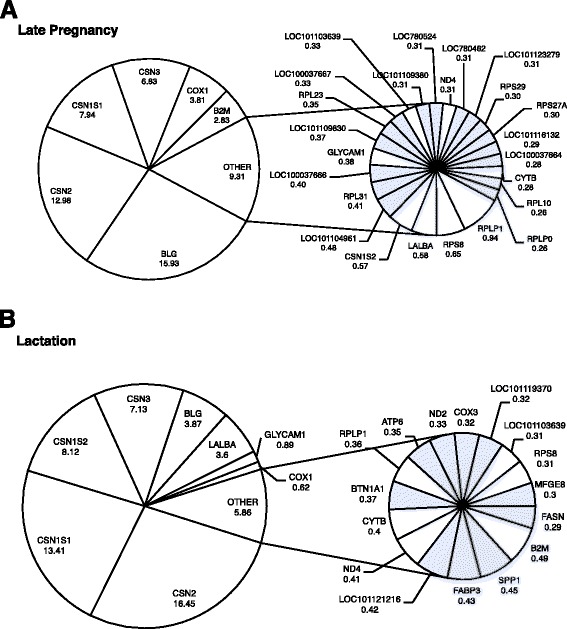
A small number of genes account for 60 % of all the RNA-seq reads. Graphical depiction of genes that were most highly expressed in the ovine mammary gland, as measured by RNA-seq, during **a**: late pregnancy (day 135 of pregnancy ± 2.4 SD, *n* = 27) and **b**: lactation (day 15 post-partum ± 1.27 SD, *n* = 18). Notes: Genes highlighted (in blue) represent genes which are highly expressed in only one physiological state. The number below each gene is the percentage of reads for that gene compared with the total number of reads in the sample

This sub-set of ultra-highly expressed genes included the major milk protein genes (caseins, α-lactalbumin and β-lactoglobulin), genes encoding ribosomal proteins, genes with products involved in energy metabolism and genes with products involved in immunity and inflammation (Fig. 1).

During late pregnancy, 18 ribosomal protein genes were ultra-highly expressed (Fig. 1) and may reflect increased levels of ribosome biogenesis in preparation for high levels of milk protein synthesis. During lactation, several metabolism-related genes were also ultra-highly expressed, which reflects the increased metabolic activity of the mammary gland [44]. Highly expressed metabolism-related genes included *COX3*, *ND2* and *ATP6*, which encode enzymes of the oxidative phosphorylation pathway, and *FASN* and *FABP3*, which are involved in milk-fat synthesis.

### Differentially expressed genes

The high expression of a small number of genes effectively dilutes the expression of other genes in the mammary gland [45] and may limit the ability to detect differences in their expression as well as introduce bias into normalization strategies. To identify differentially expressed genes analysis was performed both including and excluding these highly expressed transcripts. Although there were subtle differences to the fold-changes and RPKMs reported in the two analyses, the inclusion of the highly expressed genes did not alter the identity or number of genes identified as differentially expressed and here focus on the analysis with the highly expressed genes included. Despite this potential limitation, we were able to detect a large proportion (27 %, 2750 genes) of genes in the ovine mammary gland that were differentially expressed between late pregnancy and early lactation. A greater proportion of these genes decreased expression between late pregnancy and early lactation (1510 genes, 55 % of differentially expressed genes) while 1240 genes (45 % of differentially expressed genes) increased in expression between late pregnancy and early lactation. This trend is consistent with that of Finucane et al. [46] who used microarray analysis to examine the bovine mammary gland during lactation. Late pregnancy is a critical period for mammary development, in which the functional secretory tissue of the gland is established and differentiates prior to parturition. In sheep [24], there is limited mammary development post-partum, which may explain why the number of genes expressed in the mammary gland appears to be greater during pregnancy than in lactation. The study of Finucane et al. [46] drew parallels to small-animal models (mouse [47, 48], rat [49], and rabbit [50]), in which milk protein expression is initiated during mid-pregnancy and increases throughout pregnancy, plateauing during lactation. Thus, similarly, in sheep, the genes required for lactation may already be adequately expressed during late pregnancy.

**Fig. 2:**
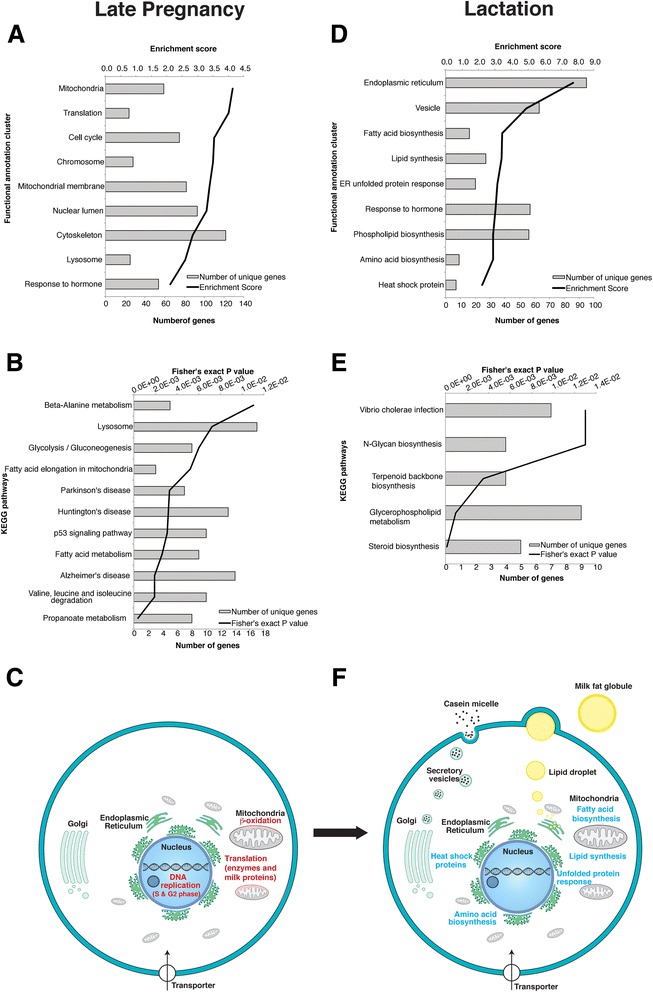
Gene ontology and KEGG pathway analysis of differentially expressed genes. Gene ontology (GO) functional annotation clusters enriched (enrichment score > 2) during **a**: late pregnancy (day 135 of pregnancy ± 2.4 SD, *n* = 27) and **d**: lactation (day 15 post-partum ± 1.27 SD, *n* = 18), and KEGG pathways enriched (Fisher’s exact *P* < 0.01) during **b**: late pregnancy and **e**: lactation in the ovine mammary gland, based on RPKM gene expression data generated by RNA-seq. The Database for Annotation, Visualisation and Integrated Discovery (DAVID) v6.7 was used to generate GO functional annotation cluster and KEGG pathway data. Summary of enriched molecular and metabolic processes in the **c**: late pregnant and **f**: lactating ovine mammary gland

Gene ontology analysis using DAVID revealed that nine functional annotation clusters (enrichment score (ES) > 2, equivalent to *P* < 0.01 [39]) and 11 KEGG pathways (*P* < 0.01) were enriched in mammary glands during late pregnancy (Figs. 2a–c and Additional files 4 and 5: Tables S5 and S7). Enriched genes were associated with gene ontology categories involved in energy metabolism (mitochondria, glycolysis, and fatty acid metabolism; in particular beta-oxidation), protein synthesis (translation initiation), cell proliferation (cell cycle, p53 signalling, chromosome and cytoskeleton) and response to hormone stimulus.

A second set of nine functional annotation clusters (ES > 2) and five KEGG pathways (*P* < 0.01) were enriched in lactating mammary glands (Figs. 2d–f and Additional files 4 and 5: Tables S6 and S8). The most enriched gene ontology categories processes were associated with lipid synthesis and secretion (endoplasmic reticulum, fatty acid biosynthesis, lipid synthesis, synthesis, and vesicle). Additionally, there was enrichment for genes associated with synthesis and post-translational processing of proteins (unfolded protein response, heat shock protein, amino acid biosynthesis).

### Expression of genes involved in cellular proliferation, survival and apoptosis in the mammary gland

**Fig. 3:**
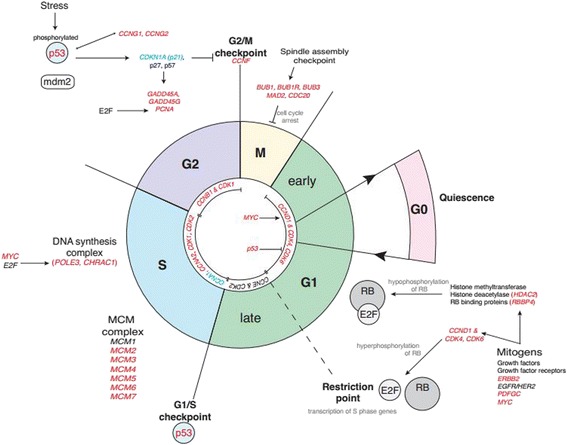
Cell cycle genes are differentially regulated between late pregnancy and lactation. Expression of cell cycle associated genes in the late pregnant (day 135 of pregnancy ± 2.4 SD, *n* = 27) and lactating (day 15 post-partum ± 1.27 SD, *n* = 18) ovine mammary gland as measured by RNA-seq. Genes shown in red were more lowly expressed while genes shown in blue were more highly expressed (RPKM *P* < 0.05, > 2-fold change in expression) during lactation, compared with late pregnancy. Genes shown in black did not have any significant change in their expression (RPKM *P* > 0.05)

It is well-known that milk production is a function of the number and activity of secretory epithelial cells [16–19], thus the mammary tissue establishment phase that occurs during pregnancy is crucial to subsequent lactation performance. Physiologically, late pregnancy is characterised by extensive structural remodelling, involving expansion of the lobulo-alveolar network, and functional differentiation of alveolar cells to secretory cells in preparation for producing milk in lactation [21, 24]. On a molecular level this is reflected in the present study through enrichment of genes associated with: regulation of cell cycle, including cyclins and cyclin-dependent kinases which form complexes to regulate cell cycle progression [51]; DNA synthesis including genes encoding the components of the MCM complex, which form part of the pre-replication complex [52], and; genes encoding histone fold proteins (*POLE3* and *CHRAC1*) (Fig. 3 and full list of genes in Additional file 5: Table S5). Also enriched were mitosis related genes, specifically those associated with the spindle assembly checkpoint, which ensures proper chromosome alignment and segregation during mitotic cell division [53, 54] (Fig. 3 and Additional file 5: Table S5). These genes are regulated by E2F family and Myc transcription factors [55]. Expression of *MYC* was higher during late pregnancy, while none of the E2F transcription factors differed in expression. However, E2F is regulated by the Retinoblastoma tumour suppressor protein (pRB), such that when pRB is hypophosphorylated it binds to E2F, inhibiting transcription of S phase target genes [56]. Genes encoding mitogenic factors known to promote hyperphosphorylation of Rb and E2F activity were more highly expressed during late pregnancy (Fig. 3), confirming E2F activity.

Given the increased DNA replication, cell division and proliferation occurring during late pregnancy, mechanisms need to be in place to account for DNA damage repair and ensure proliferation occurs in a controlled and regulated manner. The p53 signalling pathway was enriched during late pregnancy, such that expression of *TP53* was higher during lactation, as were p53-responsive DNA repair genes such as *GADD45A*, *GADD45G*, *CCNG1* and *CCNG2* (Additional file 6: Table S7). GADD45A may also use its DNA repair function to remove DNA methylation marks, potentially playing a role in epigenetic regulation of gene expression [57].

Our RNA-seq data implies that during lactation, cellular growth and proliferation processes are down-regulated, indicating that continued growth of mammary tissue is limited after parturition in sheep. These findings are consistent with transcriptomic studies in other species [46, 58, 59] and observations of cellular proliferation rates (using Ki-67) in cows, which have demonstrated a reduction in epithelial cellular expansion during lactation compared with late pregnancy [60]. In the present study we identified cell-survival markers, such as *BCL2L15*, *BCAR1*, *BAG1*, *BAG3*, *BAG5* and *BEX2*, as more highly expressed in mammary tissue during lactation compared with late pregnancy. The products of these genes may be involved in maintaining the population of milk secretory cells and milk production (galactopoiesis) during lactation, and may therefore serve as useful biomarkers or target genes for manipulation of lactation persistence.

### Expression of genes involved in energy and fat metabolism in the mammary gland

**Fig. 4:**
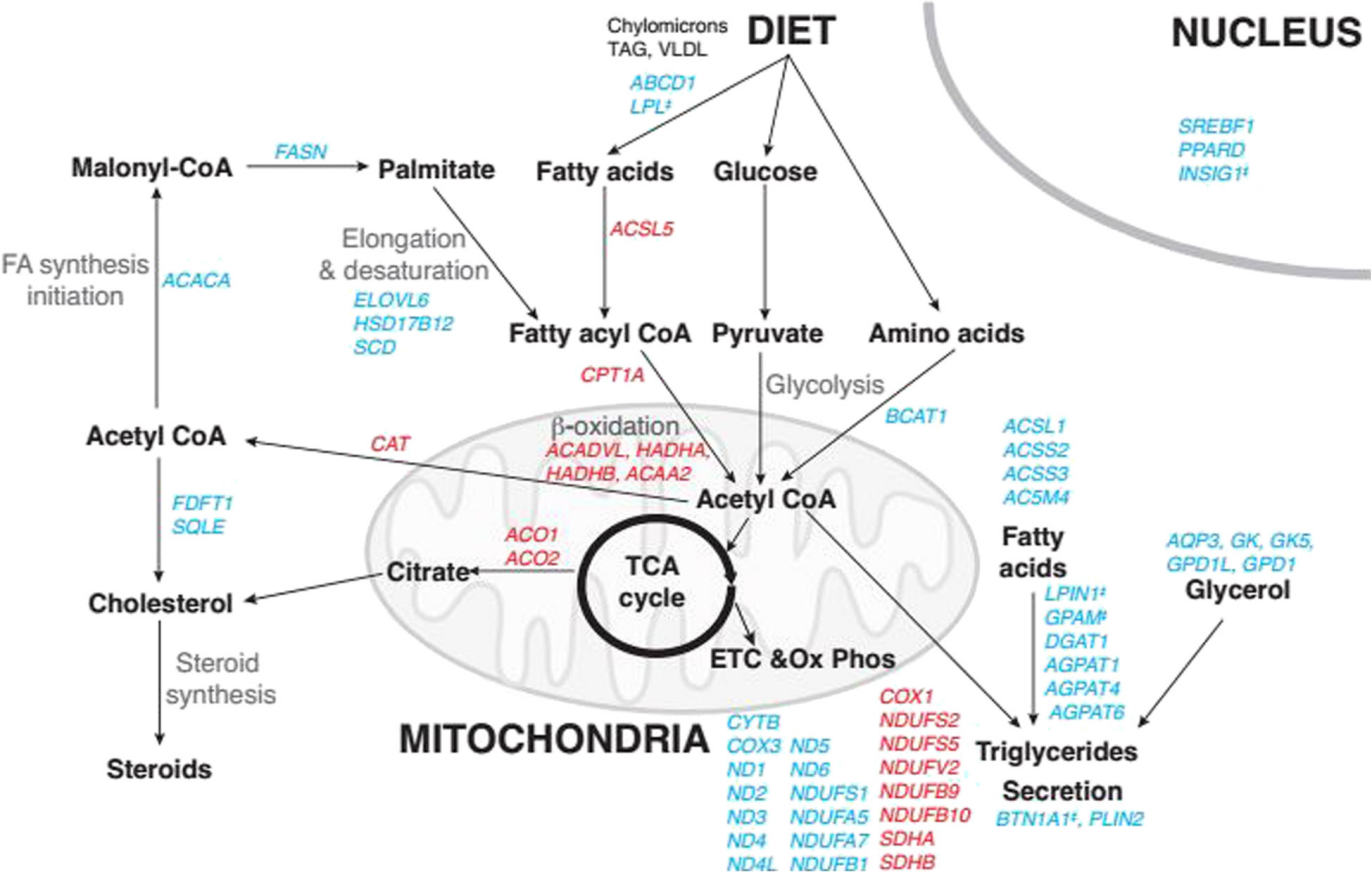
Fat metabolism genes are differentially regulated between late pregnancy and lactation. Model of the networks of genes potentially involved in the regulation of fat metabolism in the late pregnant (day 135 of pregnancy ± 2.4 SD, *n* = 27) and lactating (day 15 post-partum ± 1.27 SD, *n* = 18) ovine mammary gland, as measured by RNA-seq. Genes shown in red were more lowly expressed (RPKM *P* < 0.05, > 2-fold change in expression) during lactation, compared with late pregnancy, and were largely associated with fatty acid catabolism pathways. Genes shown in blue were more highly expressed (RPKM *P* < 0.05, > 2-fold change in expression) during lactation, compared with late pregnancy, and were largely associated with synthesis of fatty acids, triglycerides, cholesterol and steroids. Notes: double dagger denotes genes which had a >10-fold change in expression between late pregnancy and lactation

Limited growth of the mammary gland during lactation [24, 46, 60] would imply that greater activity of secretory cells is required to increase milk yield to peak lactation [19]. In support of this, we detected up-regulation of genes linked to metabolism and transport processes during lactation, consistent with studies in bovine [46] and murine [61, 62] mammary glands. In particular, genes involved in fat metabolism were enriched during lactation (full list of genes in Additional file 5: Table S6), such that there was higher expression of genes encoding enzymes involved in fatty acid biosynthesis, fatty acid activation, glycerol synthesis, triacylglycerol synthesis, cholesterol synthesis, milk fat secretion and genes involved in signal transduction of lipogenic pathways (e.g., *SREBF1*, *PPARD*, *INSIG1* and *ESR1*). Genes involved in fatty acid catabolic pathways, particularly beta-oxidation, were down regulated during lactation (Fig. 4 and Additional file 5: Table S5). These findings are consistent with the large amount of milk fat produced by the mammary gland during lactation and with findings in mice [62], pigs [59] and cows [46, 58].

Genes involved in energy metabolism, particularly genes encoding components of the mitochondrial respiratory chain, were differentially, yet highly expressed during both late pregnancy and lactation, reflecting the high-energy demands of cellular division and growth during late pregnancy, milk synthesis and secretion during lactation (Fig. 4 and Additional file 5: Tables S5 and S6). Higher expression of genes involved in amino acid biosynthesis and amino acid catabolism during lactation (Additional file 5: Table S6) is consistent with sparing of fatty acids by switching to amino acids as substrates for energy generation, as seen in bovine mammary glands [58].

### Expression of genes involved in protein synthesis in the mammary gland

**Fig. 5:**
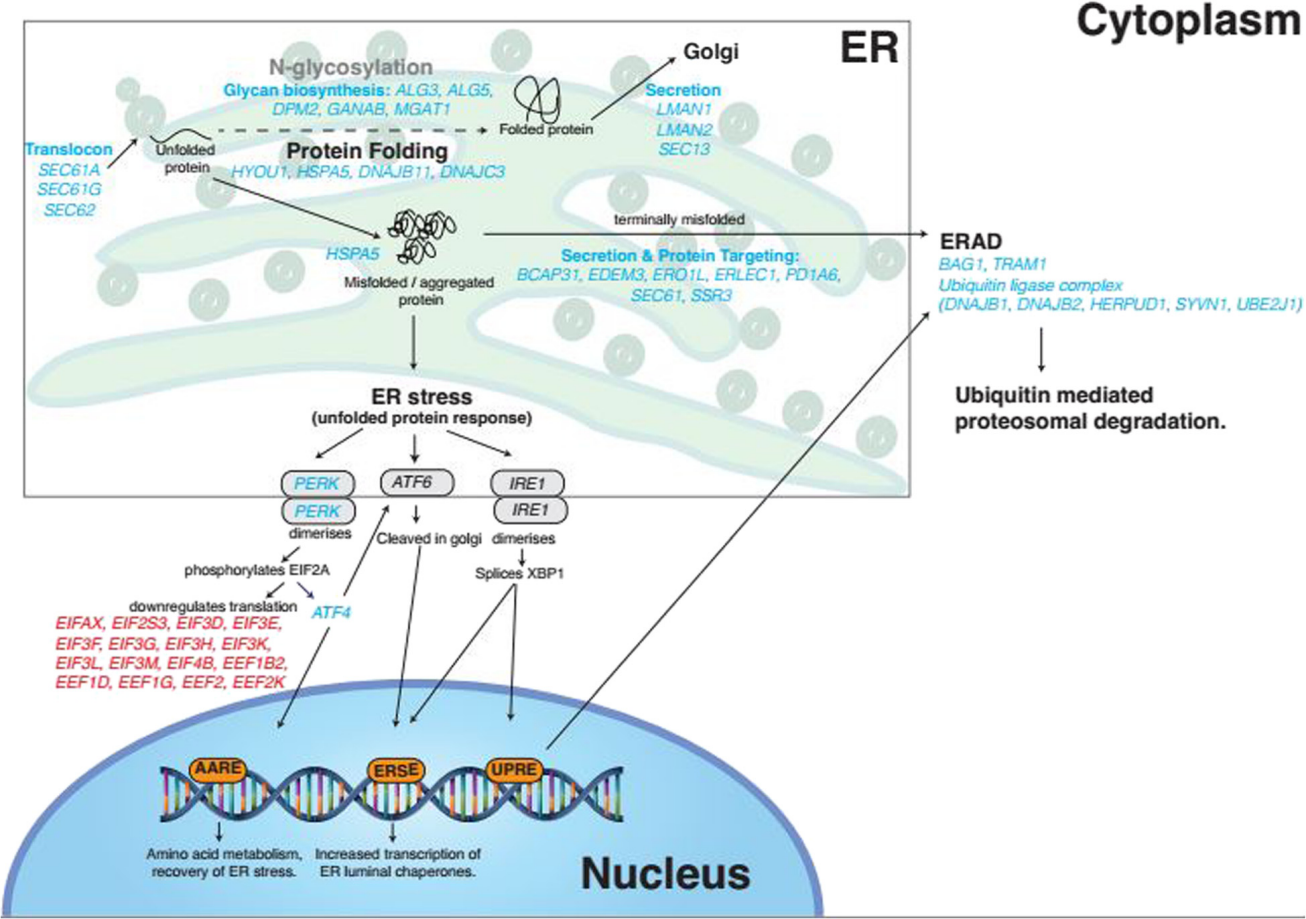
Genes involved in protein processing are differentially regulated between late pregnancy and lactation. Model of the networks of genes potentially involved in the regulation of protein processing in the late pregnant (day 135 of pregnancy ± 2.4 SD, *n* = 27) and lactating (day 15 post-partum ± 1.27 SD, *n* = 18) ovine mammary gland, as measured by RNA-seq. Genes shown in red, highlighted that protein translation was down-regulated (genes > 2-fold change in expression, RPKM *P* < 0.05) during lactation, compared with late pregnancy. Genes shown in blue highlighted that protein processing pathways, particularly the Endoplasmic Reticulum Associated Degradation (ERAD) and the unfolded protein response pathways, were enriched (genes > 2-fold change in expression, RPKM *P* < 0.05,) during lactation, compared with late pregnancy. Genes shown in black denotes no significant change in expression (RPKM *P* > 0.05) between late pregnancy and lactation

During both late pregnancy and lactation, we detected increased expression of genes associated with different aspects of protein synthesis. During late pregnancy, one of the most enriched categories of genes (functional annotation clustering enrichment score of 4.02) included genes involved in translation, translation elongation and translation initiation (Fig. 5 and Additional file 4: Table S5). Whilst the increased expression of mRNA transcripts does not necessarily reflect an increase in the levels of active protein, the large number of changes we observe in expression of genes involved in protein synthesis may reflect an increased translational requirement associated with milk proteins and enzymes being synthesised in preparation for lactation. Previous studies have also detected a decrease in expression of protein synthesis components (including genes involved in translational elongation) during lactation [43, 63]. It has been hypothesised that this down-regulation may be a mechanism for the mammary gland to prioritise translation of milk specific proteins [43] this may account for the significant changes seen in milk protein composition but only slight increases in milk protein synthesis in certain experimental regimes [64].

In rodents, the JAK2-STAT5 pathway plays an essential role in mediating expression of milk-protein genes and expression of STAT5 genes increases during lactation in response to stimulus from lactogenic hormones. In cows, however, JAK2-STAT5 signalling appears to have only a limited role in milk-protein synthesis [43]. In the present study, we detected higher expression of STAT5 genes (*STAT5A* and *STAT5B*: 1.5 and 1.4 fold, respectively, FDR *P* < 0.01) during late pregnancy, compared with lactation in sheep, which may be related to the role of STAT5 in mammary growth [29, 65, 66] as well as induction of milk-protein gene transcription. The lower expression of STAT5 during lactation, despite the dramatic increase in expression of major milk-protein genes, may indicate that, similarly to cows [43], STAT5 may not play a central role in regulating milk-protein synthesis during lactation in sheep. However, it is important to consider that regulation of STAT5 activity is predominantly through protein activation [67, 68]. Bionaz and Loor [43] suggested a possible role of STAT5 in regulating milk-protein synthesis through the E74-like factor 5 transcription factor, encoded by the *ELF5* gene. Consistent with this, expression of *ELF5* was increased during lactation (2.2 fold, FDR *P* < 0.001) in the present study, demonstrating a role of ELF5 and possibly STAT5 in transcriptional regulation of milk protein synthesis in the sheep.

Our data showed that during lactation there was also enrichment of post-translational processing of proteins, such that there was higher expression of genes in the unfolded protein response (UPR) pathway in the endoplasmic reticulum (ER) (e.g., *ATF4* transcription factor and *EIF2AK2*, also known as *PERK*) and the ER-associated degradation (ERAD) pathway, which are activated as a quality control during high levels of protein synthesis (Fig. 5). Genes encoding molecular chaperones (e.g., *HSPA8*, *HSPA5*, *DNAJB2* and *DNAJC3*) were more highly expressed in the lactating mammary gland along with genes encoding members of the HSP70 family (genes listed in Additional file 5: Table S6), which are activated at the transcriptional level by endoplasmic stress. In addition to their more traditional molecular chaperone role regulating protein folding and processing, heat shock proteins (HSPs) are also thought to have functions in immunity, inflammation and suppression of apoptosis [69]. HSPA5 and HYOU1 may function in angiogenesis in the mammary gland through promoting VEGF processing and signalling (via MAPK) inducing endothelial cell proliferation [69]. These genes may be useful targets for enhancing blood flow, and thus, nutrient supply to the mammary gland for milk production.

Genes associated with biosynthesis and transport of amino acids were more highly expressed in the ovine mammary gland during lactation, compared with late pregnancy (Additional file 5: Table S6). Supplementation of amino acids can improve milk production [70, 71] and bioavailability of amino acids, particularly lysine and methionine, and their rate of transport into MECs can be a major limiting factor for milk protein synthesis. Thus, in addition to amino acid supplementation, targeted expression and translation of amino acid transporter genes may be a useful intervention to improve milk protein synthesis and potentially promote synthesis of specific milk proteins.

### Mechanisms of transcriptional regulation: hormones and epigenetics

It is apparent that coordinated shifts in gene expression govern the switch from proliferation and differentiation to secretion in the mammary gland. These changes must be regulated by mechanisms acting at a higher level, such as endocrine and/or epigenetic regulation. The role of hormones and growth factors in the mammary gland is well known [29, 31–33], and there is growing interest in the role of epigenetics in mammary development and lactation [12, 72–74].

During both late pregnancy and lactation there was enrichment of genes associated with hormone-signalling pathways (genes listed in Additional file 4: Tables S5 and S6). Genes associated with oestrogen and corticosteroids in particular, were more highly expressed during late pregnancy compared with lactation; however, expression of genes encoding the hormones and hormone receptors themselves did not generally differ. Putative oestrogen responsive genes have been identified in the mammary gland of cattle [75], but we do not see a clear pattern of expression of these genes in our dataset; with seven out of 70 oestrogen responsive genes being more highly expressed in late pregnancy and ten out of 70 more highly expressed during lactation. This may reflect species-specific differences in genes that are responsive to oestrogen, or may reflect the complexity of oestrogen signalling to the mammary gland [31]. Similarly we did not see a clear pattern of expression associated with glucocorticoid exposure [76].

Genes associated with growth factor activity, such as IGF binding proteins two, four and seven, and V-Erb-B2 Avian Erythroblastic Leukemia Viral Oncogene Homolog 2 (*ERBB2*), which encodes a member of the epidermal growth factor (EGF) family also known as HER2, were more highly expressed during late pregnancy. Both the IGFs and EGFs are potent mitogens and have been implicated in mediating hormone effects on mammary growth [65, 77].

A number of genes encoding hormone receptors were found to be more highly expressed during lactation, e.g., oestrogen type 1 receptor (*ESR1*), thyroid hormone receptor (*ERBA BETA1*), growth hormone receptor (*GHR*), insulin receptor substrate 1 (*IRS1*), glucocorticoid receptor subfamily 3, group c, members 1 and 2 (*NR3C1* and *NR3C2*), transforming growth factor beta receptor type 1 (*TGFBR1*), as well as parathyroid hormone-related protein (*PTH*-*RP*), indicating there may be increased sensitivity of the mammary gland to these hormones during lactation. Many of these encode receptors that mediate the effects of lactogenic and galactopoietic hormones (e.g., insulin, GH and glucocorticoids) and are important for the initiation and maintenance of milk production [33, 65].

In addition to regulation of transcription by hormones, the transcriptional potential of cells in the mammary gland may be influenced by epigenetics. A sub-set of differentially expressed genes, more highly expressed during late pregnancy, were associated with chromosome and chromatin organisation (genes listed in Additional file 5: Table S5). While many of these genes may simply be related to cell division (chromosome replication and segregation) during the mammary growth phase, some may be involved in epigenetic regulation of gene expression. Alterations to the structure of chromatin can influence the recruitment of transcription factors to DNA, which may impact upon the expression of genes. Differentially expressed genes which were associated with chromatin remodelling included: *CENPA*, *H3F3A* and *H3F3B* which encode variants of the histone H3 protein, *HDAC2*, a histone deacetylase associated with transcriptional repression of genes, and *CBX3* (chromobox homolog 3), which has recently been shown to bind to gene bodies and play a role regulating genes, such as cell cycle associated genes, through transcriptional regulation, RNA processing and alternative splicing [78]. Additionally, another set of genes more highly expressed during late pregnancy were associated with RNA splicing (e.g., *HNRNPA3*, *HNRNPM*, *HNRNPF*, *HNRNPU*, *HNRNPH3*, *HNRNPD*, *HNPNPA0* and *CD2BP2*). We also identified a small, but significant (*p* = 0.03 for late pregnancy, *p* = 7.6 × 10^−3^ for lactation), number of gene clusters or “gene neighbourhoods” in our sets of differentially expressed genes (Additional file 7: Table S9). These clusters may function as chromatin domains or gene neighbourhoods that may be epigenetically regulated [79]. Collectively, these results may indicate a coordinated role for genes involved in chromatin remodelling and RNA splicing in the transcriptional regulation of the mammary gland during the transition period from late pregnancy to lactation.

## Conclusions

This is the first study on the global expression profile of the ovine mammary gland during late pregnancy and lactation. We demonstrated that 27 % of genes expressed in the ovine mammary gland are differentially expressed between late pregnancy and lactation. Our findings indicated a strong transcriptional regulation of cell proliferation, lipid metabolism and protein translation and processing, such that expression of genes involved in cell cycle, translation and fatty acid catabolism were down-regulated, while expression of genes involved in fatty acid and amino acid biosynthesis and transport, lipogenesis, and protein processing, were up-regulated during lactation. Furthermore, hormones and growth factors, signalling pathways, e.g. JAK-STAT, SREBF and PPARD, and epigenetic regulation were highlighted as having a potential key role in mediating the adaptive transcriptional changes undertaken by the ovine mammary gland to support lactation. The identification of enriched genes and pathways in the present study will provide a platform for future research into the management of mammary development, function and disease.

## Additional files

Additional file 1: Table S1. Summary of RNA-seq reads and mapping statistics for each sample. (DOCX 59 kb) Additional file 2: Table S2. RNA-seq data for genes more highly expressed during late pregnancy. Table S3: RNA-seq data for genes more highly expressed during lactation. (XLSX 1006 kb) Additional file 3: Figure S1. And supplementary text showing similarity between the RNA-seq samples using principle components analysis (PCA) and heatmaps. (DOCX 243 kb) Additional file 4:
**Supplementary text detailing RT-qPCR validation of the RNA-seq data.** **Table S4.** Candidate genes measured by RT-qPCR in late pregnant and lactating ovine mammary tissue. **Figure S2.** Correlation of RNA-seq and RT-qPCR gene expression data in the ovine mammary gland. (DOCX 213 kb) Additional file 5: Table S5. Gene ontology functional annotation clusters enriched in the ovine mammary gland during late pregnancy. **Table S6.** Gene ontology functional annotation clusters enriched in the ovine mammary gland during lactation. (XLSX 64 kb) Additional file 6: Table S7. Kyoto Encyclopaedia of Genes and Genomes (KEGG) pathways enriched in the ovine mammary gland during late pregnancy. Table S8: Kyoto Encyclopaedia of Genes and Genomes (KEGG) pathways enriched in the ovine mammary gland during lactation. (XLSX 38 kb) Additional file 7: Table S9. And supplementary text detailing the analysis used to identify clusters of differentially expressed genes in the ovine genome. (DOCX 114 kb) Additional file 8:
**Comparison of two approaches to identify differentially expressed genes during the transition from late pregnancy to lactation; CLC genomics and EdgeR.** (DOCX 137 kb)
