# Supplementary material for: Functional development of the adult ovine mammary gland—insights from gene expression profiling
Source: BMC Genomics. 2015 Oct 5;16:748. doi: 10.1186/s12864-015-1947-9 (PMC4595059; doi:10.1186/s12864-015-1947-9)
Supplement: Additional file 1: Table S1. — Summary of RNA-seq reads and mapping statistics for each sample. (DOCX 59 kb) [file 12864_2015_1947_MOESM1_ESM.docx]

## Table S1 – Summary of RNA-seq reads and mapping statistics for each sample.

| Sample ID^1^ |  | Physiological state | Total number of paired end reads | % mapping |
| --- | --- | --- | --- | --- |
| LP1 |  | Late Pregnancy | 46,564,268 | 76.19 |
| LP2 |  | Late Pregnancy | 21,764,810 | 75.63 |
| LP3 |  | Late Pregnancy | 28,494,084 | 75.82 |
| LP4 |  | Late Pregnancy | 46,964,368 | 75.79 |
| LP5 |  | Late Pregnancy | 37,729,898 | 76.11 |
| LP6 |  | Late Pregnancy | 29,126,802 | 75.66 |
| LP7 |  | Late Pregnancy | 57,023,110 | 75.25 |
| LP8 |  | Late Pregnancy | 54,432,958 | 75.93 |
| LP9 |  | Late Pregnancy | 51,655,012 | 75.10 |
| L1 |  | Lactation | 29,123,882 | 77.67 |
| L2 |  | Lactation | 35,132,680 | 84.89 |
| L3 |  | Lactation | 64,969,270 | 84.41 |
| L4 |  | Lactation | 55,143,000 | 84.77 |
| L5 |  | Lactation | 45,849,024 | 84.17 |
| L6 |  | Lactation | 32,007,694 | 84.43 |

åß

Samples consisted of pools of RNA derived from three individuals.
