# Supplementary material for: Functional development of the adult ovine mammary gland—insights from gene expression profiling
Source: BMC Genomics. 2015 Oct 5;16:748. doi: 10.1186/s12864-015-1947-9 (PMC4595059; doi:10.1186/s12864-015-1947-9)

## Additional file 3: Analysis of variation between and within treatment groups using principle components analysis (PCA) and clustered heat maps.

Principal components (PCA) and heat map analyses were performed to evaluate general patterns of variation in gene expression between treatment groups. PCA was carried out using the 5000 genes that showed the most variance in the RNA-seq analysis using the prcomp function within the R-statistical environment (Fig. S1a). Principal components (PC) analysis showed that 85.7% of the variation in gene expression between groups is explained by 2 principal components, with 83.2% of the variance explained by PC1 and only 2.5% by PC2. PC1 separates samples based on physiological state, late pregnancy and lactation. This suggests that the majority of variation in gene expression between the samples arises from the time that the mammary biopsies were collected and reflects the physiological status of the mammary gland, rather than other influences such as genetic background.

A Euclidean distance matrix was calculated between samples (calculated from log transformed total gene reads using the dist function in R) and displayed as a heatmap (Fig. S1b). This analysis demonstrates that samples within a particular physiological state (late pregnancy or lactation) are more similar to each other in terms of gene expression than comparisons between physiological states. This is consistent with the PCA analysis and suggests that the majority of variation observed in gene expression profiles is related to the physiological state of the mammary gland.

Heatmap analysis was also performed using RPKM values of the differentially expressed genes identified using CLC Genomics Workbench (Fig. S1c). This analysis demonstrates clear differences in the patterns of expression between late pregnancy and lactation, with a general trend towards very high levels of expression in late pregnancy and down regulation during lactation.

## Figure S1 – Variation between and within RNA-seq samples. A) Principle components analysis of gene expression (using the 5000 most variable genes in the RNA-seq data) in mammary biopsies obtained during late pregnancy (orange) and lactation (green). B) Heatmap based on Euclidian distances between samples. Each row is an individual sample. C) Heatmap based on similarity of gene expression profiles between samples. Each row is an individual gene included in this analysis.


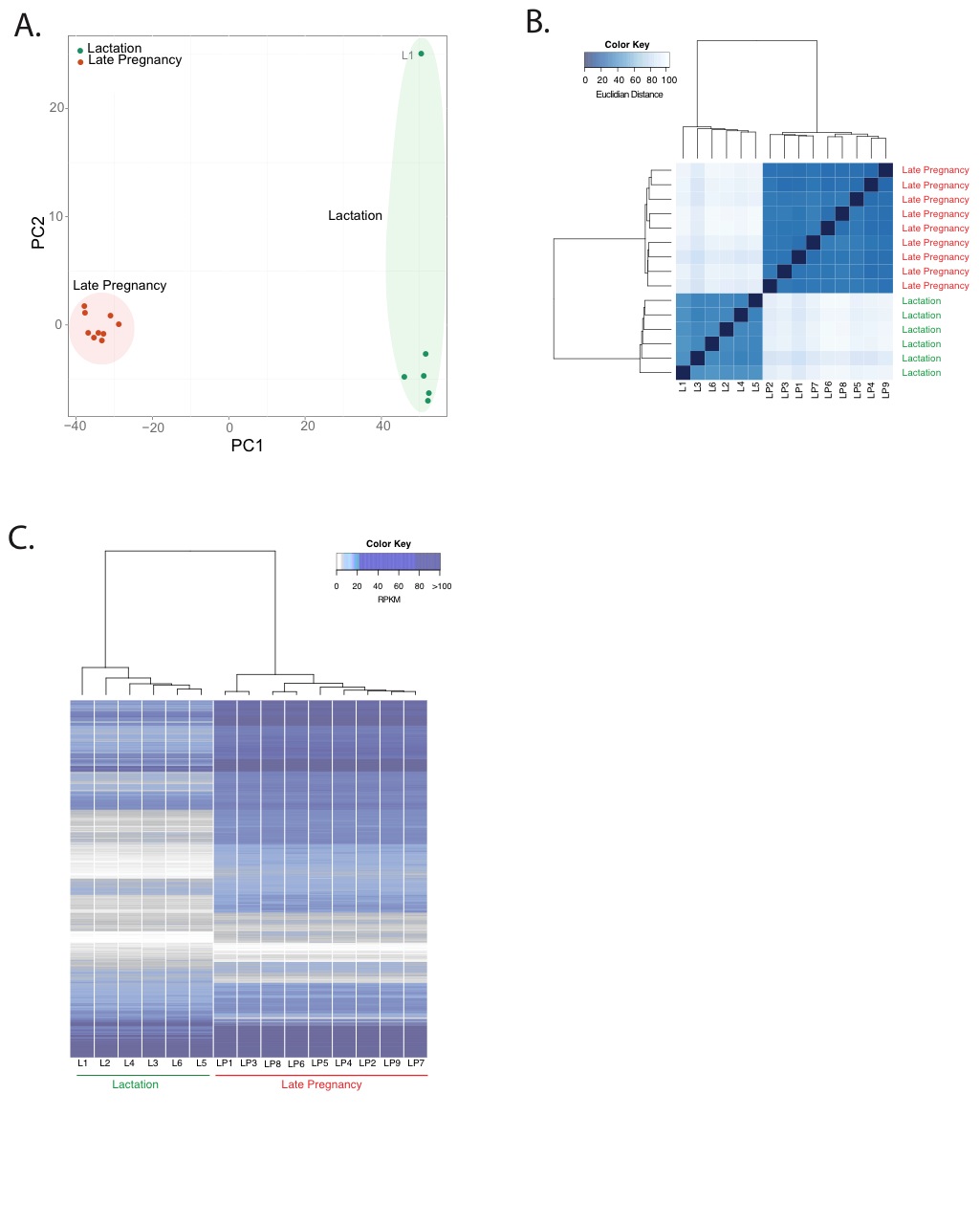

Supplement: Additional file 3: Figure S1. — And supplementary text showing similarity between the RNA-seq samples using principle components analysis (PCA) and heatmaps. (DOCX 243 kb) [file 12864_2015_1947_MOESM3_ESM.docx]
