# Supplementary material for: Functional development of the adult ovine mammary gland—insights from gene expression profiling
Source: BMC Genomics. 2015 Oct 5;16:748. doi: 10.1186/s12864-015-1947-9 (PMC4595059; doi:10.1186/s12864-015-1947-9)
Supplement: Additional file 4: — Supplementary text detailing RT-qPCR validation of the RNA-seq data. Table S4. Candidate genes measured by RT-qPCR in late pregnant and lactating ovine mammary tissue. Figure S2. Correlation of RNA-seq and RT-qPCR gene expression data in the ovine mammary gland. (DOCX 213 kb) [file 12864_2015_1947_MOESM4_ESM.docx]

## Additional file 4: Validation of differential gene expression by reverse transcription quantitative PCR (RT-qPCR)

To ensure accuracy of RNA-seq data, validation of gene expression results was conducted using reverse-transcription quantitative PCR (RT-qPCR) [1, 2]. A subset of 18 genes (Table S2) identified as differentially expressed by RNA-seq, and wide-ranging in expression levels, were confirmed by correlation with RT-qPCR analysis. Previously identified reference genes (*PRPF3* and *CUL1* [3]) were used to normalise RT-qPCR data.

RT-qPCR reactions were carried out on a Bio-Rad C1000 Thermal cycler (Bio-Rad CFX96 Real-Time System) using SsoFast EvaGreen Supermix (BioRad) with 10 x diluted cDNA template and 300 nM of oligonucleotide primers. The following PCR program was used: 1min initial incubation at 95°C, followed by 40 cycles of 5 seconds at 95°C, and 30 seconds at 60°C. On completion, the reactions were held at 95°C for 10 seconds, reduced to 65°C and incrementally raised by 0.5°C until reaching 95°C for a melt curve analysis. Reactions were carried out in duplicate for each sample to minimise effects of technical errors.

Expression levels of both the RT-qPCR and RPKM values were expressed as fold-changes for lactation relative to late pregnancy. Correlation statistics were calculated using the Spearmen and Pearson correlation models in the R statistical package [4].

There was a highly significant positive correlation 0.89 (*P* = 8.91 × 10^-7^) between gene expression data generated by RNA-seq and RT-qPCR (Figure S2), which indicates a moderate-to-strong relationship. These results demonstrate that the RNA-seq data and our analyses are sound.

## Figure S2 - Correlation of RNA-seq and RT-qPCR gene expression data in the ovine mammary gland. Graph shows the correlation of the fold changes in gene expression, between late pregnancy (day 135 of pregnancy ± 2.4 SD, n = 27) and lactation (day 15 post-partum ± 1.27 SD, n = 18), calculated for RNA-seq and RT-qPCR data.

## Table S4 – Candidate genes measured by RT-qPCR in late pregnant and lactating ovine mammary tissue.

| Gene identifier^1^ | NCBI accession | Forward primer sequence | Reverse Primer sequence | Efficiency (%) | Amplicon size (bp) |
| --- | --- | --- | --- | --- | --- |
| *FLT4* | XM_004009112.1 | CTTCCTGTCCAACCCCTTC | TAGTTTTTCCCCAACCAGCA | 98.4 | 103 |
| *FYN* | XM_004011196.1 | ATGTGGCTCCAGTTGACTCC | GTGGGTTTCCAAAGGACAAA | 95.4 | 99 |
| *JAK2* | XM_004004358.1 | AGCCTGGTGAAAGTCCCATA | TCCAAACATCTGAAGCCACA | 100.9 | 81 |
| *LALBA* | NM_001009797.1 | AAAGACGACCAGAACCCTCA | TCTTGGCACACACAATGTCA | 92.7 | 92 |
| *LPO* | NM_001009722.1 | GACAACTGCTTCCCCATCAT | CGACTGGTAAGGTGGAGTGG | 95.6 | 114 |
| *MAP4K1* | XM_004015231.1 | GGCACCTATGGGGAAGTTTT | ATCATCGTCAGGCTCCATCT | 92.8 | 90 |
| *NUMBL* | XM_004015260.1 | TGTGGATGACAAGACCAAGG | CGGCAGATGTAGGAGAAAGC | 105.9 | 113 |
| *PDGFC* | XM_004017535.1 | GGGGACTTTGTGAAGAGCAG | GCGATGGTTTCCAATCTTTC | 100.9 | 118 |
| *LOC443444* | XM_004016530.1 | GCTGGCATGGTTCTTGGA | TAGGGCTTGGCTTTCATTTG | 104.0 | 120 |
| *PTH-RP* | XM_004006757.1 | CTGGGCTGGAAGAGGACTAC | TCTGAAGGTCTCTGCTGAAAAA | 110.5 | 94 |
| *TET1* | XM_004021627.1 | TTTCTCTGGGGTCACTGCTT | TGAGCGGTTATCTTCTCGTG | 100.6 | 115 |
| *TGFBI* | XM_004008814.1 | TGGCGATGAAATCCTGGT | GGCTCCTTATTGACACTCACC | 106.4 | 117 |
| *THBS4* | XM_004010215.1 | GTTCTTGGGGCAGATGTCAC | GCATTCGGCTATGGTGTTTC | 106.8 | 109 |
| *TIMP1* | NM_001009319.2 | CCAGAATCGCAGTGAGGAGT | TCCAGGGAGCCACAAAACT | 101.9 | 89 |
| *URGCP* | XM_004018183.1 | TTATGGAGAGGGTCCGAATG | AGGCTGAGTTTCTGTGTTTGG | 93.3 | 120 |
| *VEGFC* | XM_004021836.1 | GCTGGATGTTTACAGACAAGTCC | GTAATCTGCGGGGCAAGTC | 106.0 | 100 |
| *PRPF3* | XM_004002449.1  XM_004002450.1 | ACAGATGATGGAAGCAGCAA | GGTTGGGAGGATGAAGGAGT | 101. | 105 |
| *CUL1* | XM_004008343.1 | AAAAATACAACGCCCTGGTG | CTGAGCCATCTTGGTGACTG | 116 | 95.9 |

^1^Gene symbol according to NCBI Entrez gene database http://www.ncbi.nlm.nih.gov/gene/

**References**

1. Bustin SA, Benes V, Garson JA, Hellemans J, Huggett J, Kubista M, Mueller R, Nolan T, Pfaffl MW, Shipley GL *et al*: **The MIQE Guidelines: Minimum Information for Publication of Quantitative Real-Time PCR Experiments**. *Clin Chem* 2009, **55**(4):611-622.

2. Valasek MA, Repa JJ: **The power of real-time PCR**. *Adv Physiol Educ* 2005, **29**(3):151-159.

3. Paten AM, Pain SJ, Peterson SW, Blair HT, Kenyon PR, Dearden PK, Duncan EJ: **Identification of reference genes for RT-qPCR in ovine mammary tissue during late-pregnancy, lactation and in response to maternal nutritional programming**. *Physiol Genomics* 2014.

4. Team RDC: **R: A language and environment for statistical computing**. In*.* Vienna, Austria: R Foundation for Statistical Computing; 2013.
