# Supplementary material for: Functional development of the adult ovine mammary gland—insights from gene expression profiling
Source: BMC Genomics. 2015 Oct 5;16:748. doi: 10.1186/s12864-015-1947-9 (PMC4595059; doi:10.1186/s12864-015-1947-9)
Supplement: Additional file 7: Table S9. — And supplementary text detailing the analysis used to identify clusters of differentially expressed genes in the ovine genome. (DOCX 114 kb) [file 12864_2015_1947_MOESM7_ESM.docx]

## Additional file 7: Identification of chromosomal regions that may be co-ordinately regulated during late pregnancy and lactation.

## Genes may be organised within chromosomes to form clusters or gene neighbourhoods, that may consist of functionally related genes that are co-ordinately expressed under specific conditions [[1-3](#_ENREF_1)]. It has also been proposed that coordinated expression of genes within these neighbourhoods may be mediated via epigenetic mechanisms [[4](#_ENREF_4)]. The CROC software [[5](#_ENREF_5)] was used to determine whether genes we had identified as differentially expressed were located next to each other in the sheep genome (CROC parameters used were: gene window = 5, minimum number of genes in cluster = 4, corrected *p*-value < 0.05). It is important to note that this approach is inherently limited as it only uses differentially expressed genes and cannot detect gene clusters that may be epigenetically silenced [[4](#_ENREF_4)].

## Significance of individual clusters was assessed within the CROC software correcting for multiple testing using the method of Benjamini and Hochberg [[6](#_ENREF_6)]. To determine if there were significantly more clusters than we would expect in either late pregnancy or lactation we used a permutation approach. Briefly, 10 000 simulations were performed where each simulation consisted of a random sample of the genes expressed in mammary gland consisting of the same number of differentially expressed genes in our gene list. An overall P-value was calculated using the frequency of the permutations that generated an equal or higher number of clusters than we detected in our actual dataset. For both late pregnancy and lactation we detected significantly more clusters within our list of differentially expressed genes than we might expect by chance (*p* = 0.0138 for late pregnancy, *p* = 1 × 10^-4^ for lactation*)*. This may provide support for epigenetic regulation of gene expression during the lactation cycle in sheep.

## Table S9 – Chromosomal clusters of genes identified as differentially expressed during late pregnancy and lactation.

| **Late Pregnancy** | | |
| --- | --- | --- |
| Chromosome | BH corrected *P*-value | Genes |
| NW_004080166.1 | 3.34×10^-05^ | NCF4, TEX33, TST, MPST |
| NW_004080174.1 | 4.05×10^-05^ | SRSF1, CUEDC1, VEZF1, DYNLL2 |
| NW_004080183.1 | 1.03×10^-06^ | PSMB8, TAP2, LOC101116099, LOC101109746, TAP1, PSMB9 |
| NW_004080185.1 | 1.17×10^-05^ | NSMCE4A, TACC2, PLEKHA1, HTRA1 |
|  |  |  |
| **Lactation** | | |
| Chromosome | BH corrected *P*-value | Genes |
| NW_004080166.1 | 1.24×10^-07^ | PHYHD1, CRAT, NUP188, LRRC8A, DOLK, FAM73B, SH3GLB2 |
| NW_004080168.1 | 8.61×10^-06^ | SPATA9, RHOBTB3, GLRX, ELL2 |
| NW_004080169.1 | 1.74×10^-06^ | PKD2, PPM1K, SPP1, ABCG2 |
| NW_004080183.1 | 1.08×10^-05^ | LOC101112356, LOC101105787, LOC101105290, LOC101111915, LOC101111669, LOC101113893 |
| NW_004080188.1 | 9.73×10^-07^ | SAR1A, PPA1, LRRC20, EIF4EBP2 |

**References**

1. Duncan EJ, Dearden PK: **Evolution of a genomic regulatory domain: the role of gene co-option and gene duplication in the Enhancer of split complex**. *Genome Res* 2010, **20**(7):917-928.

2. Duncan EJ, Wilson MJ, Smith JM, Dearden PK: **Evolutionary origin and genomic organisation of runt-domain containing genes in arthropods**. *BMC genomics* 2008, **9**:558.

3. Al-Shahrour F, Minguez P, Marques-Bonet T, Gazave E, Navarro A, Dopazo J: **Selection upon genome architecture: conservation of functional neighborhoods with changing genes**. *PLoS computational biology* 2010, **6**(10):e1000953.

4. Lemay DG, Pollard KS, Martin WF, Freeman Zadrowski C, Hernandez J, Korf I, German JB, Rijnkels M: **From genes to milk: genomic organization and epigenetic regulation of the mammary transcriptome**. *Plos One* 2013, **8**(9):e75030.

5. Hoijman E, Rocha-Viegas L, Kalko SG, Rubinstein N, Morales-Ruiz M, Joffe EB, Kordon EC, Pecci A: **Glucocorticoid alternative effects on proliferating and differentiated mammary epithelium are associated to opposite regulation of cell-cycle inhibitor expression**. *Journal of cellular physiology* 2012, **227**(4):1721-1730.

6. Benjamini Y, Hochberg Y: **Controlling the false discovery rate - a practical and powerful approach to multiple testing**. *J Roy Stat Soc B Met* 1995, **57**(1):289-300.
