# Supplementary material for: Functional development of the adult ovine mammary gland—insights from gene expression profiling
Source: BMC Genomics. 2015 Oct 5;16:748. doi: 10.1186/s12864-015-1947-9 (PMC4595059; doi:10.1186/s12864-015-1947-9)
Supplement: Additional file 8: — Comparison of two approaches to identify differentially expressed genes during the transition from late pregnancy to lactation; CLC genomics and EdgeR. (DOCX 137 kb) [file 12864_2015_1947_MOESM8_ESM.docx]

## Additional file 8: Comparison of two approaches to identify differentially expressed genes during the transition from late pregnancy to lactation; CLC genomics and EdgeR.

At the request of a peer reviewer request we have compared the genes identified as differentially expressed using CLC genomics with those identified using EdgeR ([*1*](#_ENREF_1)). Whilst there are qualitative differences in the genes identified (*i.e.* using EdgeR twice as many genes are identified as differentially expressed) there is also substantial overlap between the genes identified as differentially expressed using both methods (Fig. 1A) and a good correlation between the fold changes identified using both analyses (Fig. 1B). This indicates that, at least with this data set, the Baggerly test implemented in CLC genomics is more conservative than the approach taken using default parameters in EdgeR.

**Figure S3: Comparison of genes identified as differentially expressed using CLC genomics and EdgeR.** (A) number of DGEs identified using EdgeR and CLC Genomics workbench. (B) correlation between fold changes reported for DEGs using EdgeR and CLC Genomics Workbench.


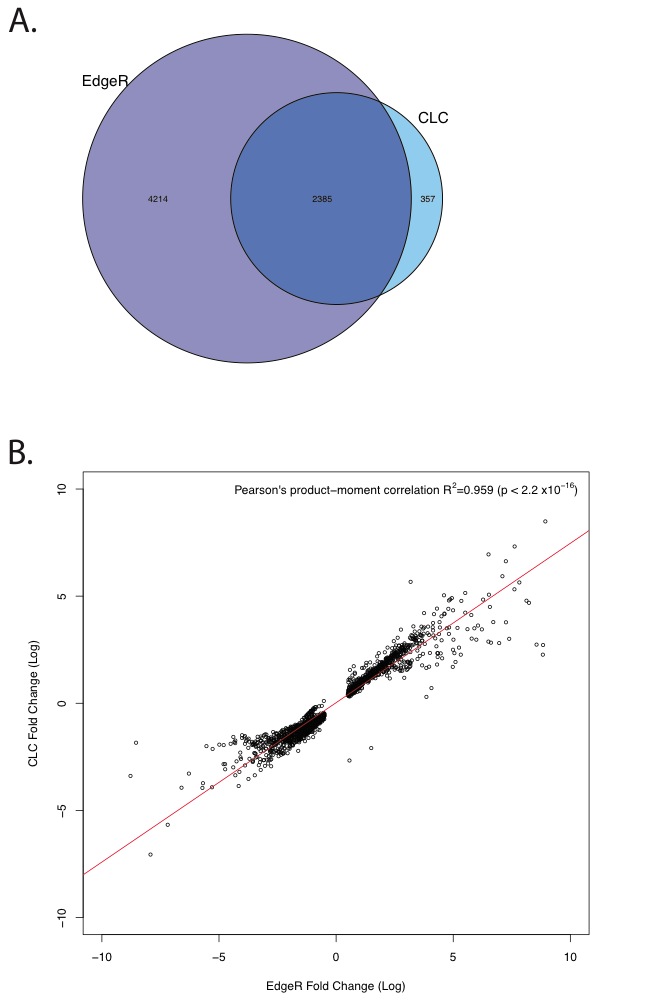


1. M. D. Robinson, D. J. McCarthy, G. K. Smyth, edgeR: a Bioconductor package for differential expression analysis of digital gene expression data. *Bioinformatics* **26**, 139 (Jan 1, 2010).
